# Tissue-specific isoform switch and DNA hypomethylation of the pyruvate kinase PKM gene in human cancers

**DOI:** 10.18632/oncotarget.1159

**Published:** 2013-08-07

**Authors:** Shruti Desai, Minming Ding, Bin Wang, Zhimin Lu, Qi Zhao, Kenna Shaw, W.K. Alfred Yung, John N. Weinstein, Ming Tan, Jun Yao

**Affiliations:** ^1^ Mitchell Cancer Institute, Departments of Cell Biology and Neuroscience, University of South Alabama, Mobile, USA; ^2^ Division of Biostatistics, School of Public Health, The University of Texas – Houston Health Science Center, Houston, USA; ^3^ Department of Genetics, The University of Texas M. D. Anderson Cancer Center, The University of Texas – Houston Health Science Center, Houston, USA; ^4^ Department of Neuro-Oncology, The University of Texas M. D. Anderson Cancer Center, The University of Texas – Houston Health Science Center, Houston, USA; ^5^ Ludwig Collaborative Laboratory, Department of Neurosurgery, Johns Hopkins University, Baltimore, USA.; ^6^ Department of TCGA Genome Data Analysis Center, The University of Texas M. D. Anderson Cancer Center, The University of Texas – Houston Health Science Center, Houston, USA; ^7^ Department of Bioinformatics and Computational Biology, The University of Texas M. D. Anderson Cancer Center, The University of Texas – Houston Health Science Center, Houston, USA

**Keywords:** PKM2, alternative splicing, DNA methylation

## Abstract

The M2 isoform of pyruvate kinase (PKM2) plays an important role in aerobic glycolysis and is a mediator of the Warburg effect in tumors. It was previously thought that tumor cells switch expression of PKM from normal tissue-expressed PKM1 to tumor-specific PKM2 via an alternative splicing mechanism. This view was challenged by a recent report demonstrating that PKM2 is already the major PKM isoform expressed in many differentiated normal tissues. Here, through analyses on sixteen tumor types using the cancer genome atlas RNA-Seq and exon array datasets, we confirmed that isoform switch from PKM1 to PKM2 occurred in glioblastomas but not in other tumor types examined. Despite lacking of isoform switches, PKM2 expression was found to be increased in all cancer types examined, and correlated strongly to poor prognosis in head and neck cancers. We further demonstrated that elevated PKM2 expression correlated well with the hypomethylation status of intron 1 of the PKM gene in multiple cancer types, suggesting epigenetic regulation by DNA methylation as a major mechanism in controlling PKM transcription in tumors. Our study suggests that isoform switch of PKM1 to PKM2 in cancers is tissue-specific and targeting PKM2 activity in tumors remains a promising approach for clinical intervention of multiple cancer types.

## INTRODUCTION

Aerobic glycolysis, wherein cells take up more glucose and produce large amounts of lactate in presence of oxygen, is a key feature of many cancer cells. This altered glucose metabolism is known as Warburg effect [1]. Though this altered glucose metabolism is less efficient in ATP production it is thought to increase metabolic intermediates that are required for synthesis of biological macromolecule [1,2]. Accumulating evidence suggests that many oncogenes and tumor suppressors (e.g. c-Myc, ErbB2, and p53) can affect Warburg effect on different regulation levels [3-5]. Studies also identified that mTOR, a rapamycin target, could induce HIF1 which increases glycolysis in cells [6,7]. In addition, PKM2, the M2 isoform of the glycolytic enzyme pyruvate kinase, has been shown to promote Warburg effect and tumorigenesis by functioning as a HIF1 co-activator [8-11]. Increased activity of PKM2 in human cancers is reported to regulate amino acid homeostasis and affect protein expression in thyroid cancer [12].

Pyruvate kinase (PK) is a rate limiting glycolytic enzyme that converts phosphoenolpyruvate and ADP to pyruvate and ATP. PKM1 and PKM2 are alternatively spliced products of the same primary RNA transcript, encoded by the *PKM* gene. The two isoforms differ at 23 amino acid residues as PKM1 mRNA contains exon 9 and lacks exon 10, whereas PKM2 mRNA contains exon 10 and lacks exon 9 [13]. The alternative splicing of PKM2 mRNA transcript is reported to be mediated by members of heterogeneous nuclear ribonucleoprotein (hnRNP) family which are regulated by c-Myc [14].

In addition to its role as a pyruvate kinase, PKM2 seems to have other nuclear functions that are independent of its function in cancer cell metabolism. For example, PKM2 can function as a protein kinase to phosphorylate histone H3 and promote tumorigenesis [15]. PKM2, but not PKM1, is translocated into nucleus after EGFR activation and binds beta-catenin to regulate gene transcription [16]. Elevated expression of PKM2 has been reported in many types of cancers including colon and breast cancers [17,18] and is associated with poor prognosis in signet ring cell gastric cancer and esophageal squamous cell cancer [19,20]. As such, efforts were made to identify small molecule inhibitors specifically targeting PKM2 for anticancer therapy [21].

PKM2 is often considered as the predominant isoform expressed in highly proliferative and cancer cells [21-23]. However, a recent study used mass spectrometry to demonstrate that there is no evidence for the exchange of PKM1 to PKM2 expression during cancer formation [24]. In this study, we performed a complete survey of PKM2 status in sixteen tumor types using the cancer genome atlas (TCGA) RNA-Seq and exon array datasets. We demonstrate that PKM2 isoform switch in cancers is tissue-specific and only occurred in glioblastomas. In addition, we demonstrate that overexpression of PKM2 in most tumor types is regulated by hypomethylation of intron 1 near the *PKM* gene promoter.

## RESULTS

### Expression levels of PKM1 and PKM2 in human cancers

**Figure 1:**
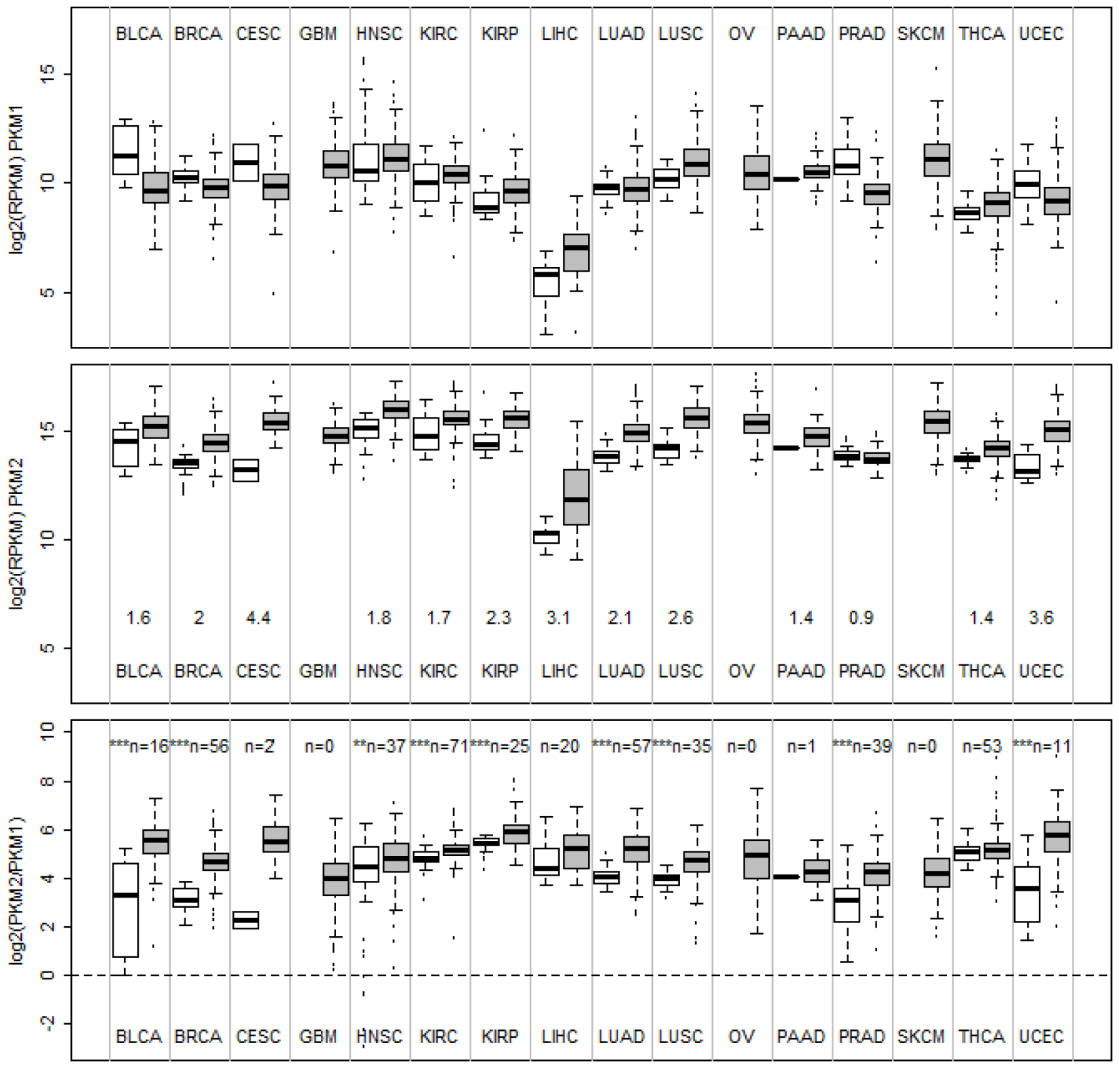
Boxplots of mRNA expression of PKM1, PKM2, and PKM2/PKM1 ratios in normal tissues and human cancers mRNA expression data of PKM1 and PKM2 for various cancers and autologous normal controls were obtained from TCGA RNA-SeqV2 level 3 datasets as logged RPKM (see materials and methods) and plotted. BLCA, bladder carcinoma, BRCA, breast invasive carcinoma, CESC, cervical squamous cell carcinoma, GBM, glioblastoma, HNSC, head and neck squamous carcinoma, KIRC, kidney renal clear cell carcinoma, KIRP, kidney renal papillary carcinoma, LIHC, liver hepatocellular carcinoma, LUAD, lung adenocarcinoma, LUSC, lung squamous carcinoma, OV, ovarian serous cystadenocarcinoma, PAAD, pancreatic adenocarcinoma, PRAD, prostate adenocarcinoma, SKCM, skin cutaneous melanoma, THCA, thyroid carcinoma, UCEC, endometrial carcinoma. Number of autologous normal controls were labeled in the bottom panel (n=). Statistically significant changes in PKM2/PKM1 ratio between normal tissue and cancers were marked by asterisks (**, *p*<0.01, ***, *p*<0.001 by two sample *t*-test).

**Figure 2:**
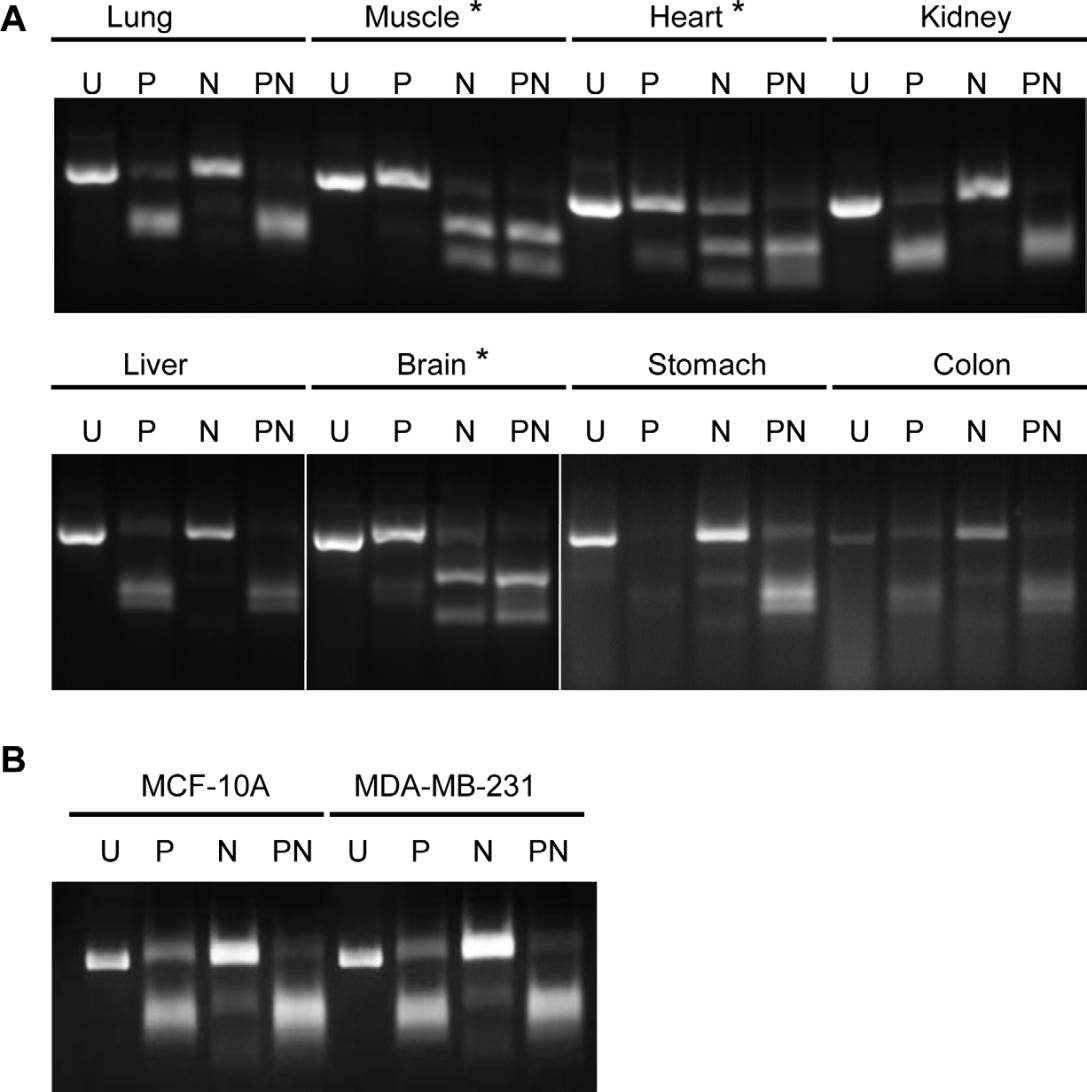
Determination of major PKM isoforms in normal tissues using RT-PCR and restriction enzyme digestion Total RNA was analyzed by RT-PCR followed by enzymatic digestion with PstI (P), NcoI (N) or both enzymes (PN) and an uncut control (U) in (A) normal tissues and in (B) breast cell lines. Samples with a clear PKM2 digestion pattern (muscle, heart, and brain) are labeled with asterisks (see methods [25]).

To examine PKM1 and PKM2 expression levels in human tumors and in normal tissues, we analyzed the level 3 RNA-Seq data from the TCGA datasets, which contain normalized RPKM values (Reads Per Kilobase per Million mapped reads) for each isoform of all genes (see methods and materials for details). The results on sixteen different types of cancers are summarized by boxplots in Figure 1. Overall, PKM1 expression is much lower than that of PKM2 in both normal and tumor tissues examined, with a median logged RPKM around 10 compared to a median logged RPKM of nearly 15 for PKM2. Liver has the least PKM expression among all tissues examined. PKM2 expression is elevated in almost all tumor types examined compared to autologous normal tissues except for prostate cancer. The changes of median PKM2 mRNA expression from normal control to tumor samples range from 1.4 fold in thyroid cancer to more than 3 fold in endometrial, liver, and cervical cancers. For some tumor types (glioblastoma, ovarian cancer, and melanoma) normal control was not available. Among all tumors having normal controls we found PKM2 was expressed as the major isoform in both tumor and normal tissues. We further examined the PKM2/PKM1 ratio in both normal and tumor tissues (Figure 1, bottom panel). This revealed that PKM2/PKM1 ratio increased in majority of the tumors compared to autologous normal samples, suggesting there might be a further shift of expression toward the PKM2 isoform in tumors. To validate our *in silico* analysis of TCGA RNA-Seq data, we performed experiments to amplify PKM cDNA from multiple human tissues using RT-PCR followed by restriction enzyme digestion to estimate PKM1 and PKM2 composition [25]. As shown in Figure 2A, this confirmed that tissues other than muscle, heart, and brain all express mainly PKM2 rather than PKM1. PKM2 is also the major expressed isoform in a breast cancer cell line MDA-MB-231 and a non-tumorigenic breast epithelial cell line MCF-10A (Figure 2B). In addition, results from Illumina Body Map project also confirmed that PKM2 is the major isoform in most of the tissues except for skeletal muscle, heart, and brain (Figure S1).

### Differential PKM1 to PKM2 isoform switches in glioblastomas subtypes

**Figure 3:**
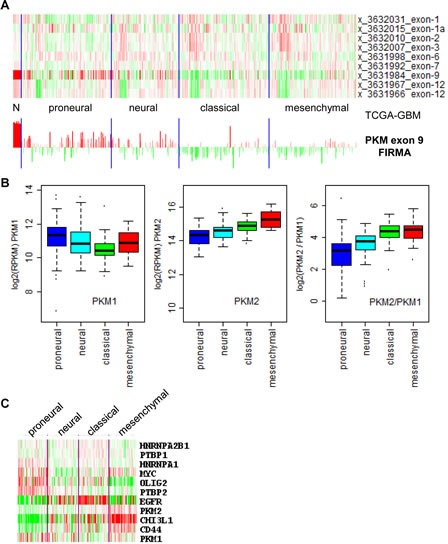
Differential PKM1 to PKM2 isoform switch in glioblastoma (GBM) subtypes (A), top, heatmap of PKM2 exon array FIRMA values for normal brain (N) and GBMs (in subtypes as labeled); bottom, barplot of FIRMA values from -5 to 7 of the exon array probe covering PKM exon 9 (PKM1-specific exon). (B), boxplots of mRNA expression of PKM1, PKM2, and PKM2/PKM1 ratios in GBM subtypes. mRNA expression data of PKM1 and PKM2 for GBM subtypes were obtained from TCGA RNA-SeqV2 level 3 datasets as logged RPKM values (see material and methods). (C), heatmap of mRNA expression of PKM genes, their known regulators, and glioblastoma subtype markers. Data were obtained from TCGA RNA-SeqV2 level 3 datasets as logged RPKM with median removal and drawn by the TreeView software.

Since the TCGA RNA-SeqV2 did not have normal controls for glioblastoma and ovarian cancer, we next examined TCGA exon array data where normal control were available for both tumors. The level 3 data for exon array were in an output format named FIRMA, which can be considered as a normalized score measuring whether an exon is differentially expressed among a set of samples (in this case, a combination of normal and tumor samples) [26]. As shown in Figure 3A, all normal brain samples had the highest positive FIRMA values for the PKM1-specific exon (exon 9), whereas in glioblastomas exon 9 FIRMA values were much lower and some were negative, suggesting isoform switch from PKM1 to PKM2 in these tumors. Unfortunately, the exon array did not cover the PKM2-specific exon (exon 10), so a direct evaluation of exon 10 usage between normal brain and glioblastoma could not be undertaken. Unlike normal brain, normal ovarian samples did not show elevated exon 9 FIRMA values, suggesting no isoform shift toward PKM2 in ovarian cancers (supplemental Figure S2). In addition, we observed different degrees of PKM2 isoform switch among glioblastoma molecular subtypes, with “proneural”-type glioblastoma having low degree of PKM2 switch and “classical” and “mesenchymal” glioblastomas having higher degree of PKM2 isoform switch. This was confirmed by the TCGA RNA-SeqV2 data, showing higher PKM1 expression in proneural glioblastomas and higher PKM2 expression in classical and mesenchymal glioblastomas. The authenticity of the glioblastoma subtypes used in this study is supported by the result in Figure 3C, where subtype-specific expression of OLIG2, EGFR, CD44, and CHI3L1/YKL-40 were seen as expected. Unexpectedly, MYC and PTB genes were high in proneural glioblastomas where PKM1 but not PKM2 was highly expressed. Thus, combined with results from Figure 1 and 2, we confirmed PKM2 isoform switch within brain tumors but not in other tumors despite that a minor shift of ratio toward more PKM2 expression was observed in nearly all tumors.

### Hypomethylation of PKM gene intron 1 correlated with elevated PKM expression in tumors

**Figure 4:**
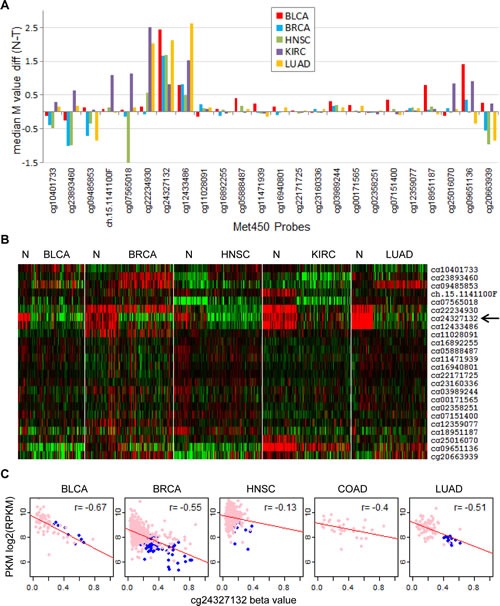
Identification of hypermethylation sites in the PKM gene corresponding to reduced PKM expression (A), differential methylation of the PKM gene between cancers and normal controls. Differences of median M values between various tumors and autologous normal controls were plotted for all Met450 probes covering the PKM gene. (B), heatmap of M values for various tumors and autologous normal controls. Methylation M values for PKM genes were collected from up to 50 normal controls along with 100 randomly selected tumors and clustered after median removal. Red, hypermethylation, green, hypomethylation. (C), correlation of PKM intron 1 hypermethylation to reduced PKM expression. DNA methylation beta value for probe cg24327132 (located in intron 1, figure S4) were plotted against PKM expression data from RNA-seq in multiple cancers. Pink, tumors, blue, normal control. Pearson correlation r values were labeled.

Since PKM2 expression was elevated in human tumors, we examined whether this could be due to changes in methylation status of the PKM gene. TCGA level 3 DNA methylation data were analyzed and results from five tissue types including bladder, breast, head and neck, kidney, and lung in which both normal and tumor data were available were shown in Figure 4A, where median differential methylation M values between normal and tumor tissues were plotted for all 24 methylation probes covering the PKM gene. This identified the probe cg24327132 as the most differentially methylated probe between normal and tumor in five tissues. A heatmap showing M value differences of the PKM gene between various normal and tumor tissues also suggested that cg24327132 and the nearby cg12433486 were two probes consistently hypermethylated in normal tissues and became demethylated in tumors (Figure 4B). The locations of these probes are inside intron 1 of PKM2 gene, which are 3095bp and 2023bp downstream of the transcriptional start site (supplemental Figure S3). Comparing methylation data with PKM expression using data from RNA-Seq (combined PKM1 and PKM2 expression) revealed strong negative correlation between PKM expression and cg24327132 methylation status in bladder, breast, colon, and lung cancers (Figure 4C), having a Pearson coefficient ranging from -0.4 to -0.67. On the other hand, this correlation was weak in head and neck cancer (Pearson r -0.13) and kidney cancer (data not shown), despite significant differences in cg24327132 methylation between normal and tumor tissues, suggesting additional mechanisms might be involved in regulating PKM expression other than cg24327132 methylation in these tumors. Thus, decreased methylation in intron 1 of PKM gene near its promoter may contribute to increased PKM expression in tumor tissues.

### PKM2 expression is a poor prognostic factor in head and neck cancer

**Figure 5:**
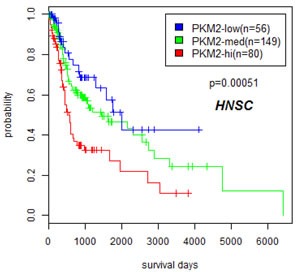
Overexpression of PKM2 correlated with poor prognosis in head and neck cancers Kaplan Meier plot is drawn on patients stratified by K means (k=3) method to divide head and neck cancer patients into PKM2-low, medium, and high expressing groups using data from TCGA.

Since PKM2 expression was elevated in multiple cancer types, we asked whether overexpression of this important molecule in cancer metabolism correlated with patient outcome. We used Cox proportional hazard regression to examine relationship between PKM2 expression and overall patient survival in various tumor types. Out of 16 cancer types examined, only in head and neck cancer we observed a strong correlation of PKM2 expression to overall patient survival, with a Cox regression *p* value of 7.78e-5. Kaplan Meier analysis (Figure 5) using Kmeans method to divide patient into PKM2-high, medium, and low expressing groups further indicated that patients bearing PKM2-high tumors had worst prognosis and patients bearing PKM2-low tumors had favorable prognosis, consistent with the role of PKM2 in tumor progression. Thus, PKM2 is unlikely to have prognostic values in most human cancers examined except for head and neck cancer.

## DISCUSSION

It is thought and often quoted in literature that PKM1 is found in many normal differentiated tissues, whereas PKM2 is expressed in most proliferating cells, including in all cancer cell lines and tumors [21-23]. However, experimental data supporting such claims especially data from primary human tumors are lacking. A recent study from Bluemlein et al using mass spectrometry to precisely determine PKM1 and PKM2 protein levels provided clear evidence that PKM2 instead of PKM1 is found in many normal differentiated tissues, therefore there is no isoform shift from PKM1 toward PKM2 in most of tumors [24]. Here we performed an extensive study on 16 tumor types using the TCGA RNA-Seq datasets to examine mRNA levels of PKM1 and PKM2 in both normal and tumor tissues. We found that indeed PKM2 is the major expressed isoform in most of the normal differentiated tissues except for muscle and brain, confirming previous findings made by Bluemlein et al. However, we observed a consistent shift toward more PKM2 expression in almost all tumors. The reason for this in many cases small shift is not clear. One possibility is that normal tissues used in this study are heterogeneous, and contain components such as fibroblasts or muscle-related cells (e.g. myoepithelial cells) which expressed more PKM1 [24], and tumor tissues may contain less numbers of these types of cells. We cannot exclude the possibility that tumor cells may turn on programs to maximize PKM2 production despite that PKM2 is already the major isoform expressed in corresponding normal tissues.

Although there is no major PKM2 isoform switch in most of the tumors examined, we did confirm that such a switch happened in glioblastomas, in which corresponding normal tissues express PKM1. In addition, we found that degrees of PKM2 switch in glioblastoma subtypes differ, with proneural glioblastomas having the least PKM2/PKM1 ratio, and mesenchymal glioblastomas the highest PKM2/PKM1 ratio. This is consistent with the fact that proneural glioblastomas are thought to be more differentiated and mesenchymal glioblastoma more undifferentiated. We were unable, however, to confirm increased MYC and PTB gene expression in PKM2-high tumors, as previously reported [14].

Despite infrequent PKM2 isoform switch in most of the tumors, we found increased PKM2 expression in nearly all tumors compared to their normal counterparts, suggesting overexpression of PKM2 may play a common tumor-promoting role in all cancers. We further identified that hypomethylation of sites within intron 1 of the PKM gene correlated extremely well with PKM gene expression, suggesting epigenetical regulation of PKM gene by DNA methylation may be a major mechanism to cause PKM overexpression in tumors. Note, this overexpression of PKM is not isoform specific at the transcription level. It is the following specific splicing steps in conjunction to increased transcription to cause PKM2 but not PKM1 overexpression in cancers.

Previously it has been reported that PKM2 behaves as a poor prognostic factor in signet ring cell gastric cancer and esophageal squamous cell cancer [19,20]. These tumors types were not included in our study. Among 16 tumor types examined, we found that PKM2 acts as a strong poor prognostic factor only in head and neck cancers. In addition, in glioblastomas four samples with the highest PKM2 expression are among the samples with poorest survival (data not shown), although Cox proportional hazard regression analysis in glioblastoma did not yield statistically significant output. These results suggest that despite its known importance in cancer metabolism, PKM2 is not generally linked to patient outcome.

In summary, our results reconciled previous findings on PKM2 isoform switch in tumors and demonstrate that PKM2 isoform switch in cancer is tissue-specific. Isoform switch of PKM2 in glioblastomas, overexpression of PKM2 in most tumor types via hypomethylation near the PKM gene promoter, and its prognostic value in head and neck cancer justify the development of PKM2 inhibitors as promising anti-cancer therapeutics.

## METHODS

### Molecular profiling datasets and data preprocessing

Level 3 RNA-SeqV2 data (containing data on gene, isoform, exon, and junction levels), level 3 exon array FIRMA data, level 3 Agilent microarray gene expression data, level 2 DNA methylation data (Infinium Human Methylation 450), and clinical data for multiple cancers were downloaded from The Cancer Genome Atlas (TCGA) data portal (https://tcga-data.nci.nih.gov/tcga/dataAccessMatrix.htm). For DNA methylation data, M values were calculated as log2 ratio of methylated intensity over unmethylated intensity [27].

### Determination of PKM1 and PKM2 expression levels in TCGA datasets

Based on PKM gene structure (supplemental Figure S3) and refgene exon information we assigned isoforms uc002att.1, uc002atv.1, uc002atw.1, uc002atx.1, uc010ukj.1, uc010ukk.1, and uc010bit.1 to PKM1 and isoforms uc002atr.1, uc002ats.1, uc002atu.1, uc002aty.1, uc010biu.1, uc010uki.1, and uc002atz.1 to PKM2. RNA-SeqV2 data for these isoforms were obtained from TCGA and summed up to give PKM1 and PKM2 expression levels.

### Determination of PKM1 and PKM2 isoform expression using RT-PCR

2μg of RNA was used from different tissue samples obtained from Origene (Rockville MD). Reverse transcriptase was carried out using High Capacity cDNA reverse transcription kit from Applied Biosystems. Semi-quantitative PCR was carried out using GoTaq-green master mix (Promega, Madison WI) and PKM primers as described earlier [25]. The amplified product was divided into four aliquots and digested with PstI (P) only, NcoI (N) only, both (PN) or neither (U). The products were analyzed on a 3% agarose gel with ethidium bromide.

### Clustering analysis and statistical testing

Clustering analyses were done using the Cluster and TreeView software (available at http://rana.lbl.gov/EisenSoftware.htm). K-means grouping, Cox proportional hazard regression, and Kaplan-Meier log rank test were done using the R software (http://www.r-project.org).

## SUPPLEMENTARY MATERIAL AND FIGURES



## References

[1] Warburg O (1956). On the origin of cancer cells. Science.

[2] Hsu PP, Sabatini DM (2008). Cancer cell metabolism: Warburg and beyond. Cell.

[3] Dang CV, Kim JW, Gao P, Yustein J (2008). The interplay between MYC and HIF in cancer. Nat Rev Cancer.

[4] Zhao YH, Zhou M, Liu H, Ding Y, Khong HT, Yu D, Fodstad O, Tan M (2009). Upregulation of lactate dehydrogenase A by ErbB2 through heat shock factor 1 promotes breast cancer cell glycolysis and growth. Oncogene.

[5] Jiang P, Du W, Wang X, Mancuso A, Gao X, Wu M, Yang X (2011). p53 regulates biosynthesis through direct inactivation of glucose-6-phosphate dehydrogenase. Nat Cell Biol.

[6] Demidenko ZN, Blagosklonny MV (2011). The purpose of the HIF-1/PHD feedback loop: to limit mTOR-induced HIF-1alpha. Cell Cycle.

[7] Leontieva OV, Blagosklonny MV (2011). Yeast-like chronological senescence in mammalian cells: phenomenon, mechanism and pharmacological suppression. Aging (Albany NY).

[8] Christofk HR, Vander Heiden MG, Harris MH, Ramanathan A, Gerszten RE, Wei R, Fleming MD, Schreiber SL, Cantley LC (2008). The M2 splice isoform of pyruvate kinase is important for cancer metabolism and tumour growth. Nature.

[9] Hitosugi T, Kang S, Vander Heiden MG, Chung TW, Elf S, Lythgoe K, Dong S, Lonial S, Wang X, Chen GZ, Xie J, Gu TL, Polakiewicz RD, Roesel JL, Boggon TJ, Khuri FR (2009). Tyrosine phosphorylation inhibits PKM2 to promote the Warburg effect and tumor growth. Sci Signal.

[10] Luo W, Hu H, Chang R, Zhong J, Knabel M, O'Meally R, Cole RN, Pandey A, Semenza GL (2011). Pyruvate kinase M2 is a PHD3-stimulated coactivator for hypoxia-inducible factor 1. Cell.

[11] Luo W, Semenza GL (2011). Pyruvate kinase M2 regulates glucose metabolism by functioning as a coactivator for hypoxia-inducible factor 1 in cancer cells. Oncotarget.

[12] Bluemlein K, Gluckmann M, Gruning NM, Feichtinger R, Kruger A, Wamelink M, Lehrach H, Tate S, Neureiter D, Kofler B, Ralser M (2012). Pyruvate kinase is a dosage-dependent regulator of cellular amino acid homeostasis. Oncotarget.

[13] Noguchi T, Inoue H, Tanaka T (1986). The M1- and M2-type isozymes of rat pyruvate kinase are produced from the same gene by alternative RNA splicing. J Biol Chem.

[14] David CJ, Chen M, Assanah M, Canoll P, Manley JL (2010). HnRNP proteins controlled by c-Myc deregulate pyruvate kinase mRNA splicing in cancer. Nature.

[15] Yang W, Xia Y, Hawke D, Li X, Liang J, Xing D, Aldape K, Hunter T, Alfred Yung WK, Lu Z (2012). PKM2 phosphorylates histone H3 and promotes gene transcription and tumorigenesis. Cell.

[16] Yang W, Xia Y, Ji H, Zheng Y, Liang J, Huang W, Gao X, Aldape K, Lu Z (2011). Nuclear PKM2 regulates beta-catenin transactivation upon EGFR activation. Nature.

[17] Eigenbrodt E, Basenau D, Holthusen S, Mazurek S, Fischer G (1997). Quantification of tumor type M2 pyruvate kinase (Tu M2-PK) in human carcinomas. Anticancer Res.

[18] Luftner D, Mesterharm J, Akrivakis C, Geppert R, Petrides PE, Wernecke KD, Possinger K (2000). Tumor type M2 pyruvate kinase expression in advanced breast cancer. Anticancer Res.

[19] Lim JY, Yoon SO, Seol SY, Hong SW, Kim JW, Choi SH, Cho JY (2012). Overexpression of the M2 isoform of pyruvate kinase is an adverse prognostic factor for signet ring cell gastric cancer. World J Gastroenterol.

[20] Zhan C, Shi Y, Lu C, Wang Q (2013). Pyruvate kinase M2 is highly correlated with the differentiation and the prognosis of esophageal squamous cell cancer. Dis Esophagus.

[21] Vander Heiden MG, Christofk HR, Schuman E, Subtelny AO, Sharfi H, Harlow EE, Xian J, Cantley LC (2010). Identification of small molecule inhibitors of pyruvate kinase M2. Biochem Pharmacol.

[22] Mazurek S, Boschek CB, Hugo F, Eigenbrodt E (2005). Pyruvate kinase type M2 and its role in tumor growth and spreading. Semin Cancer Biol.

[23] Wong N, De Melo J, Tang D (2013). PKM2, a Central Point of Regulation in Cancer Metabolism. Int J Cell Biol.

[24] Bluemlein K, Gruning NM, Feichtinger RG, Lehrach H, Kofler B, Ralser M (2011). No evidence for a shift in pyruvate kinase PKM1 to PKM2 expression during tumorigenesis. Oncotarget.

[25] Clower CV, Chatterjee D, Wang Z, Cantley LC, Vander Heiden MG, Krainer AR (2010). The alternative splicing repressors hnRNP A1/A2 and PTB influence pyruvate kinase isoform expression and cell metabolism. Proc Natl Acad Sci U S A.

[26] Purdom E, Simpson KM, Robinson MD, Conboy JG, Lapuk AV, Speed TP (2008). FIRMA: a method for detection of alternative splicing from exon array data. Bioinformatics.

[27] Du P, Zhang X, Huang CC, Jafari N, Kibbe WA, Hou L, Lin SM (2010). Comparison of Beta-value and M-value methods for quantifying methylation levels by microarray analysis. BMC Bioinformatics.

