# Supplementary material for: Tissue-specific isoform switch and DNA hypomethylation of the pyruvate kinase PKM gene in human cancers
Source: Oncotarget. 2013 Aug 7;5(18):8202–10. doi: 10.18632/oncotarget.1159 (PMC4226677; doi:10.18632/oncotarget.1159)
Supplement: Supplementary file 1 [file oncotarget-05-8202-s001.pdf]

Tissue-specific isoform switch and DNA hypomethylation of the pyruvate kinase PKM gene in human cancers – Desai et al

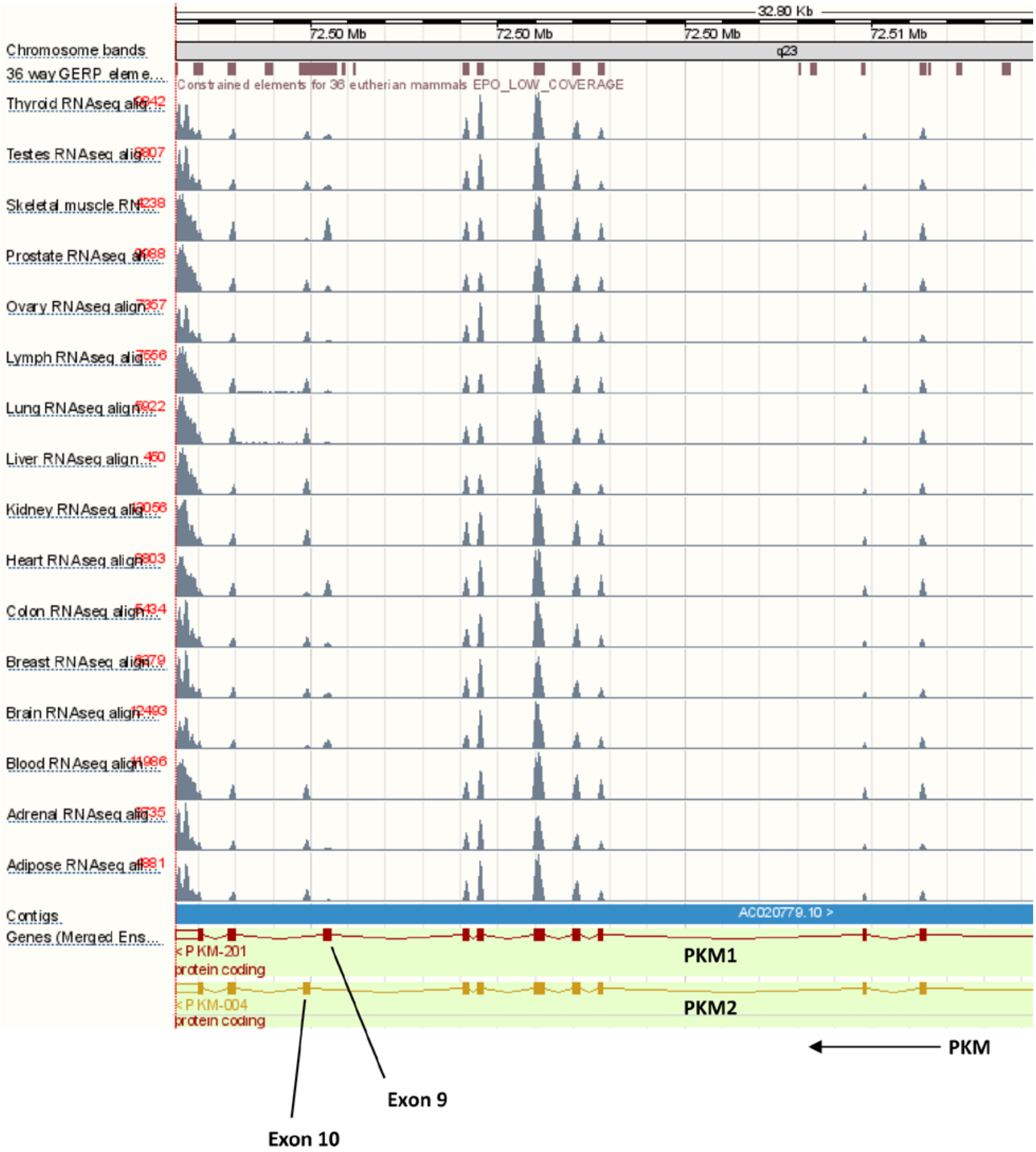

Figure S1: Expression of PKM1/2 exons in normal tissues, data from the Illumina Body Map project.

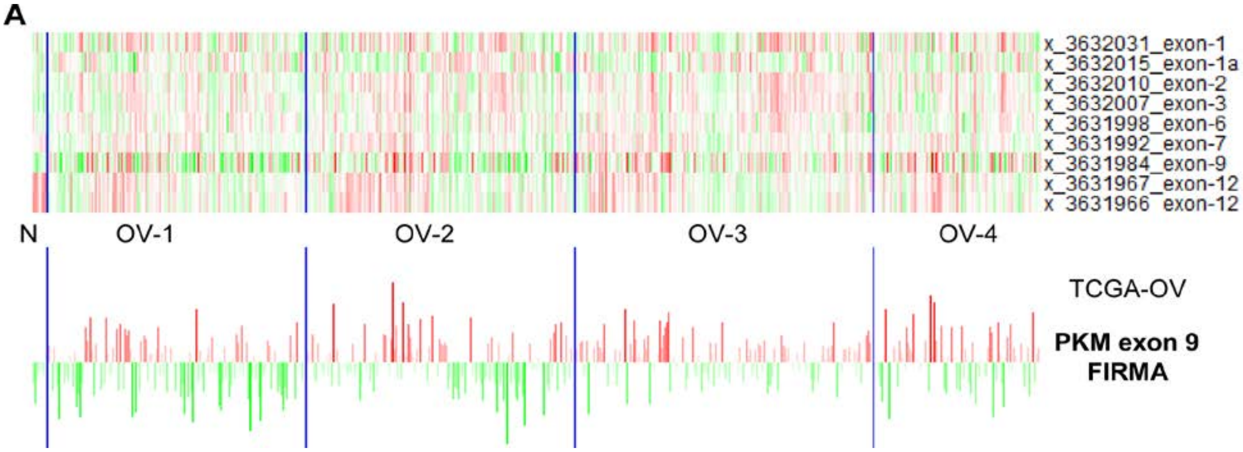

Figure S2: Heatmap of exon array FIRMA values in ovarian cancers and lung squamous carcinomas along with normal controls.

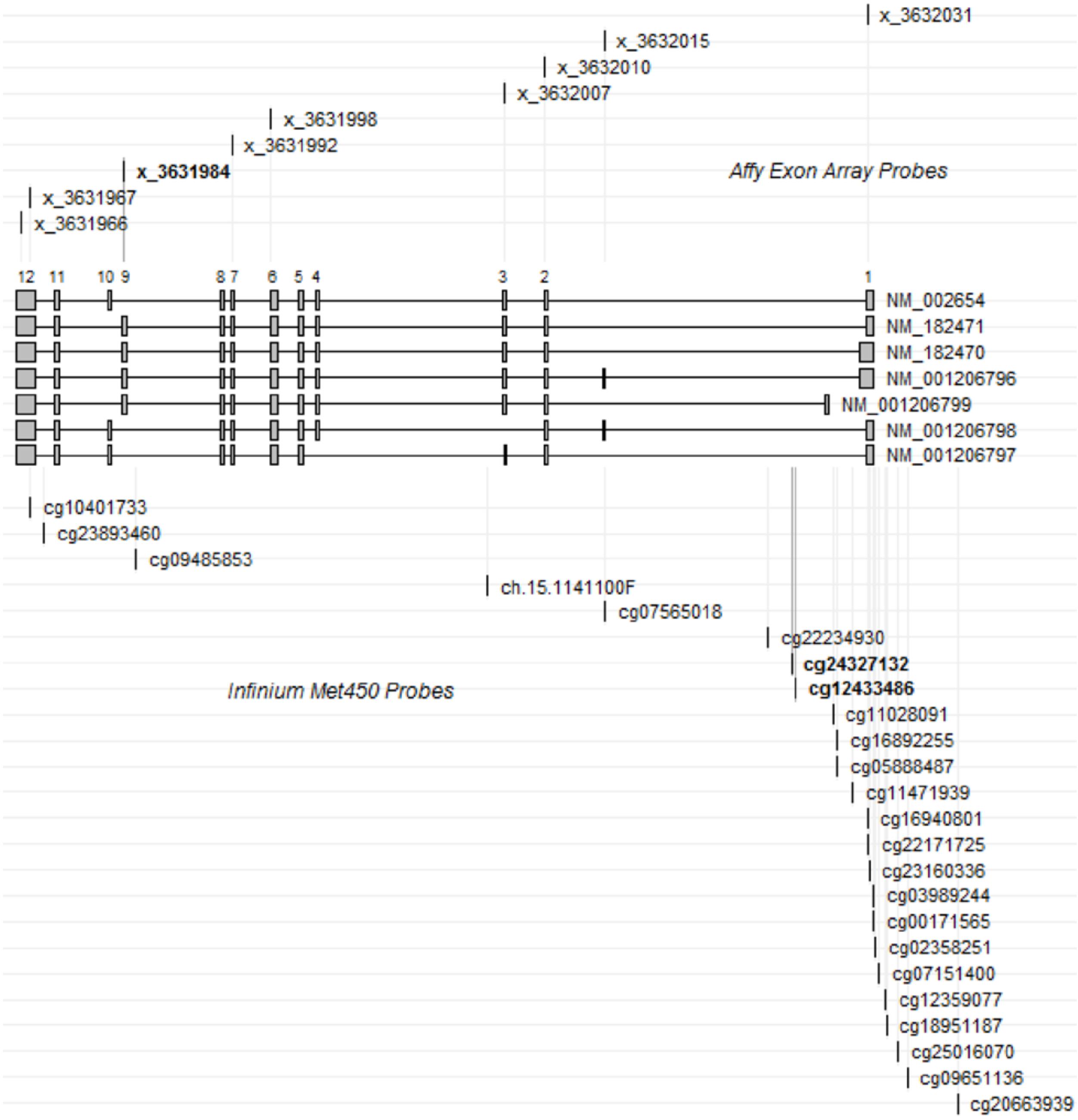

Figure S3: PKM gene structure and positions of exon array probes and Infinium Met450 methylation probes. The exon array probe within PKM1 exon9 and two probes (cg24327132 and cg12433486) mostly related to PKM expression were highlighted.
